# Supplementary material for: Genetic ablation of serotonin receptor 2B improves aortic valve hemodynamics of Notch1 heterozygous mice in a high-cholesterol diet model
Source: PLoS One. 2020 Nov 25;15(11):e0238407. doi: 10.1371/journal.pone.0238407 (PMC7688160; doi:10.1371/journal.pone.0238407)
Supplement: S1 Fig — 1320 mm/s is the 95th percentile of peak velocity found in ref. [33]. Using this criteria, 33%, 25%, and 12.5% of wild-type, knockout heterozygous, and knockout null mice developed hemodynamic CAVD, respectively. (DOCX) [file pone.0238407.s001.docx]

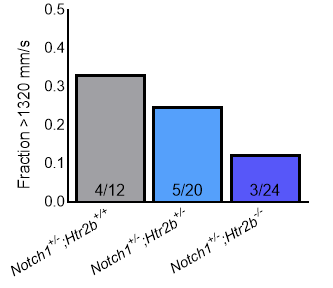


**S1 Fig. Portion of mice with hemodynamic CAVD based on criteria from (33).** 1320 mm/s is the 95^th^ percentile of peak velocity found in ref. (33). Using this criteria, 33%, 25%, and 12.5% of wild-type, knockout heterozygous, and knockout null mice developed hemodynamic CAVD, respectively.
