# Supplementary material for: Genetic ablation of serotonin receptor 2B improves aortic valve hemodynamics of Notch1 heterozygous mice in a high-cholesterol diet model
Source: PLoS One. 2020 Nov 25;15(11):e0238407. doi: 10.1371/journal.pone.0238407 (PMC7688160; doi:10.1371/journal.pone.0238407)
Supplement: S3 Fig — Randomly selected samples from wild-type, knockout, DMSO, and SB204741 groups were selected and RT-qPCR was performed on the Htr2a gene. Expression was very low and there was no significant change. Mean+/-SE, *P<0.05, Mann-Whitney U-test. (DOCX) [file pone.0238407.s003.docx]

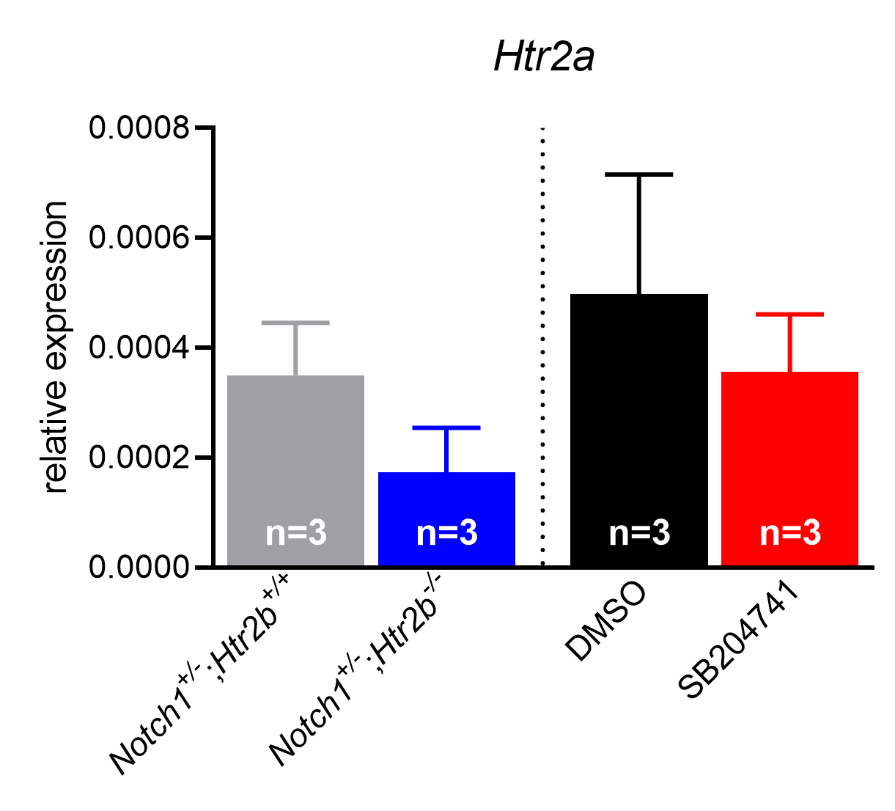


Gene: *Htr2a*, F: aaccccattcaccatagcc, R: tgccacaaaagagcctatgag

**S3 Fig. Genetic ablation or pharmacological inhibition did not result in compensation of other 5-HT receptor subtypes.** Randomly selected samples from wild-type, knockout, DMSO, and SB204741 groups were selected and RT-qPCR was performed on the *Htr2a* gene. Expression was very low and there was no significant change. Mean+/-SE, *P<0.05, Mann-Whitney U-test.
