# Supplementary material for: Genetic ablation of serotonin receptor 2B improves aortic valve hemodynamics of Notch1 heterozygous mice in a high-cholesterol diet model
Source: PLoS One. 2020 Nov 25;15(11):e0238407. doi: 10.1371/journal.pone.0238407 (PMC7688160; doi:10.1371/journal.pone.0238407)
Supplement: S4 Fig — Statistical comparison is difficult due to wide variability in tissue characteristics in A. wild-type and B. DMSO treatment groups. (DOCX) [file pone.0238407.s004.docx]

**
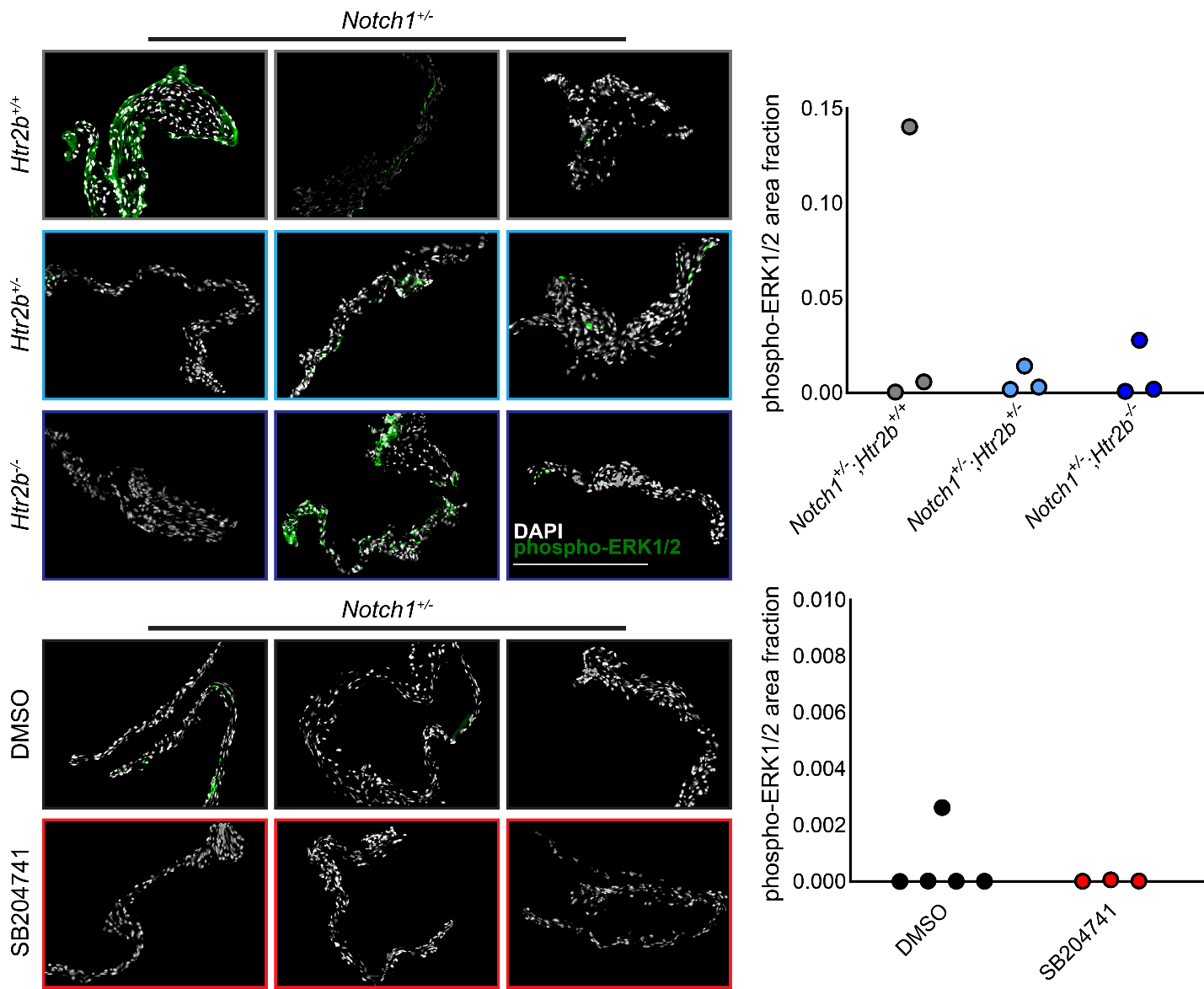
**

**S4 Fig. High phospho-ERK1/2 stain variability in *Notch1^+/-^;Htr2b^+/+^* and DMSO groups.** Statistical comparison is difficult due to wide variability in tissue characteristics in **A.** wild-type and **B.** DMSO treatment groups.
